# Evolution of self-sustained circadian rhythms is facilitated by seasonal change of daylight

**DOI:** 10.1098/rspb.2022.0577

**Published:** 2022-11-30

**Authors:** Motohide Seki, Hiroshi Ito

**Affiliations:** ^1^ Faculty of Design, Kyushu Univesity, Fukuoka 815-8540, Japan; ^2^ Institute for Asian and Oceanian Studies, Kyushu University, Fukuoka 819-0395, Japan

**Keywords:** circadian rhythms, evolution, self-sustained, gene regulatory networks

## Abstract

Self-sustained oscillation is a fundamental property of circadian rhythms and has been repeatedly tested since the early days of circadian research, resulting in the discovery of almost all organisms possessing self-sustained circadian oscillations. However, the evolutionary advantage of self-sustainability has been only speculatively discussed. In this theoretical study, we sought the environmental constraints and selection pressure that drive the acquisition or degeneration of self-sustainability through the process of evolution. We considered the dynamics of a gene regulatory network having a light input pathway under 12 h light and 12 h dark cycles or multiple day length conditions and then optimized the network structure using an evolutionary algorithm. By designing the fitness function in the evolutionary algorithm, we investigated the environmental conditions that led to the evolution of the self-sustained oscillators. Then, we found that self-sustained oscillation is rarer than damped oscillation and hourglass-type behaviour. Moreover, networks with self-sustainability have a markedly high fitness score when we assume that a network has to generate a constantly periodic expression profile regardless of day length. This study is, to our knowledge, the first to show that seasonality facilitated the evolution of the self-sustained circadian clock, which was consistent with empirical records.

## 1. Introduction

Circadian rhythms are repetitive physiological phenomena with a period of 24 h. Three unique properties of circadian rhythms—self-sustained oscillations, temperature compensation of the oscillation period, and entrainment by diurnal light or temperature cycles—are shared across kingdoms [1]. Self-sustained oscillation, that is, maintaining the oscillation amplitude without any periodic stimulations, is generally regarded as a more fundamental property than the other two. Temperature compensation assumes self-sustainability because it claims that the period of self-sustained circadian rhythm can be constant under different temperatures. The theory of entrainment argues that external cycles can entrain self-sustained oscillations as long as the applied force is sufficiently strong to shift the phase of the oscillator. Thus, these two properties postulate that the circadian clock system possesses self-sustainability.

**Figure 1.**
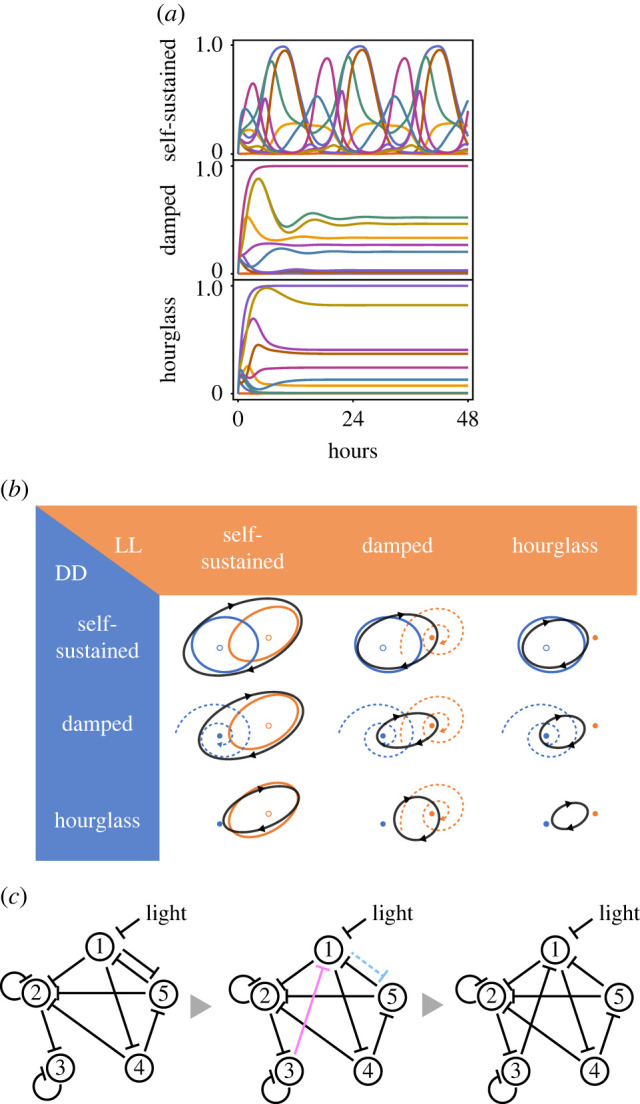
Procedure of evolutionary calculation and classification of systems. (*a*) Examples of gene expression dynamics obtained under constant darkness. Based on the dynamics, systems are classified into three categories: self-sustained oscillators (top panel), damped oscillators (middle panel) and hourglasses (bottom panel). (*b*) Classification of systems based on the dynamics under light and dark (LD) cycles. Systems are classified according to their behaviour under constant darkness (DD, blue) or constant light exposure (LL, orange) into three categories: self-sustained oscillators, damped oscillators and hourglasses. The three-by-three classes are possible under LD cycles, where the system approaches a closed orbit (black), different from the orbits under LL or DD. (*c*) Illustration of evolutionary calculation. The parent (left panel) has five nodes and 10 inhibitory relationships in this example. One randomly chosen inhibitory relationship (coloured cyan in the middle panel) was removed and one inhibitory relationship (coloured in magenta) was randomly added. Thus, the offspring (right panel) had the same number of inhibitory relationships as the parents.

Self-sustainability in the circadian rhythms of model organisms has been repeatedly tested since the early days of circadian research. For example, the circadian rhythms of leaf movement last for at least one week under dark conditions, where the environmental time cues are completely shut out [2]. Rodents exhibit circadian rhythms in locomotor activity, a physiologically fundamental measure of circadian rhythm, for several months under constant darkness [3]. However, a few studies have reported two groups of organisms lacking self-sustained circadian oscillations, yeast [4], aphids [5] and purple bacteria [6] are examples of the first group. These organisms possess damped circadian oscillators, indicating that their oscillation amplitudes diminish after being released under constant conditions. Cyanobacteria *Prochlorococcus* and *Hydra vulgaris* exhibited another type of loss of self-sustainability. After these organisms are transferred to constant conditions, the expression levels do not exhibit any peaks and promptly reach an equilibrium state. However, these organisms can sense environmental light and respond to light-dark (LD) diurnal cycles [7,8]. Hereafter, we precisely distinguish the systems that lose self-sustainability using ‘damped oscillator’ for the system that exhibits damping oscillations under constant conditions, such as former examples and ‘hourglass’ for the just-photosensitive system like the latter cases (figure 1*a*,*b*). This classification theoretically corresponds to a stable spiral and a stable node in the trajectory around a fixed point in nonlinear dynamics [9].

Most daily rhythms that have ever been reported satisfy the criteria of self-sustainability, and thus, they are regarded as bona fide circadian oscillations. However, even a damped oscillator and hourglass can show forced oscillations under LD cycles, as long as the light signal can affect these semi-clock systems. Thus, regardless of the self-sustainability of the system, the systems behave similarly in a diurnally cyclic environment. This fact brings the question: why has self-sustainability evolved under periodic daily conditions on Earth? Cyanobacteria possess three clock genes *kaiA*, *kaiB* and *kaiC* [10], and these clock genes were acquired in the order of *kaiC*, *kaiB* and *kaiA* [11]. In addition, the null mutant of *kaiA*, that is, the strain with only *kaiB* and *kaiC*, shows damped oscillation [12], suggesting that the cyanobacterial circadian clock evolved from a damped oscillator to a self-sustained oscillator.

The advantage of self-sustainability has been only speculatively discussed since the early days of circadian research [13]. One can say that developing an endogenous self-sustained oscillator allows the organism to anticipate and prepare daily events to react properly. Self-sustained oscillators maintain their phase as an indicator of their internal state. If circadian machinery has a system that outputs a signal depending on the phase of its clock, the system can measure the time elapsed from the onset or offset of light signals [14]. However, damped oscillators and hourglasses also can exploit this advantage. An hourglass contains system variables that converge to an equilibrium state. Suppose an hourglass system connects with the output system that responds to decay of a variable in hourglass. In that case, the hourglass-type system measures the elapsed time from the switching of environmental light intensity. In principle, a damped oscillator can take advantage of both the phase and decay information if the system can separate these values.

Another hypothesis is seasonality. If the system wants to know the exact time in a day, it is not sufficient to measure the elapsed time from the change in light intensity because the time of sunrise and sunset depends on the season. Thus, a season-independent clock requires additional machinery to absorb the variation in day length. Self-sustained oscillators naturally incorporate an absorber because the entrained phase of the circadian clock depends on the day length; that is, a well-tuned phase response to light stimulation enables day length-free behaviour. However, a damped oscillator or hourglass-type system can infer the time of day if the system incorporates machinery that preprocesses light signals and then absorbs seasonal variation of photoperiod. Thus, the advantages of the self-sustainability of the circadian clock remain unknown.

This study investigated the environmental constraints and selection pressures that drive the acquisition or degeneration of self-sustainability through evolution. By designing the fitness function of the evolutionary algorithm that leads to self-sustained oscillations, we investigated the environmental conditions under which a self-sustained oscillator is more advantageous.

Several theoretical studies have attempted to explain the evolution of the molecular network of circadian rhythms. For example, Roenneberg & Merrrow [15] considered a coupled system comprising five feedback loops. Each feedback system exhibits damped oscillations under constant conditions; on the other hand, it does forced oscillations under LD cycles. When the strength of the coupling between the feedback loops increases, the system shows self-sustained oscillation, even under constant conditions, suggesting that this type of alteration in parameters occurred during the evolution of the circadian clock. Troein *et al.* [16] used an evolutionary algorithm to search for gene regulatory networks (GRNs) in which gene expression is enhanced just after dawn and just before dusk. They concluded that more complex GRNs were better adapted to seasonal changes in the photoperiod or unpredictable noisy environments. From not the evolution of circadian rhythms but a purely theoretical context, Kobayashi *et al.* [17] proposed an evolutionary algorithm to find oscillatory GRNs with a specific period length. They also showed that the oscillation period could be easily tuned by modifying the regulations in the optimized GRN.

In this study, we employed a modified version of Kobayashi’s model and used an evolutionary algorithm to examine the evolutionary scenarios of self-sustained oscillations. In particular, considering that there are rarely pure constant light and dark conditions on Earth, we designed a fitness based on the dynamics under LD cycles; that is, we did not explicitly consider self-sustainability under constant conditions for the calculation of fitness, but searched for the conditions in which a self-sustained oscillator behaves more properly than a damped oscillator and hourglass. Through this evolutionary computational search, we tackled the old question in chronobiology: what drives the evolution of self-sustained oscillation?

## 2. Methods

### (a) The model for gene regulatory network

We extended the model of Kobayashi *et al.* [17] by incorporating an input pathway for the light signal (figure 1*c*). The original model considers a GRN consisting of *N* genes (labelled as gene 1, gene 2,…and gene *N*) and *M* inhibitory links (*M* ≤ *N*^2^). The model does not implement direct activation links, while it captures indirect activation relationships through a chain of an even number of inhibitory links. For example, a chain of two links, a direct inhibitory link from gene A to gene B and one from B to C, result in an indirect activation link from A to C. Note that a direct activation link from A to C is well-approximated by this indirect link via B especially when B has no links other than the above-mentioned two links. In addition, each gene is allowed to have a link with itself, namely, direct autoinhibition. Every GRN is represented by a corresponding binary matrix $A=(a_{ij})$, where *a*_*ij*_ = 1 if the expression of gene *i* is inhibited by gene *j* and *a*_*ij*_ = 0 otherwise. Note that A can represent a complex of two or more independent GRNs. We identified such a subdivisible matrix (see appendix A) and excluded it from the analyses below.

In the present study, we analysed a case in which the gene expression 1 was inhibited by light. The light signal was defined as a square wave function of time *t* toggling between 0 (dark) and 1 (light), which was approximated by a continuous function *L*(*t*) in numerical computations to avoid computational errors (see appendix B). Note that we can consider the case in which the gene expression 1 is inhibited under dark conditions by simply changing the interpretations of *L*(*t*) = 0 and *L*(*t*) =1.

Expression level of gene *i* at time *t* is denoted by *U*_*i*_(*t*). Dynamics of gene expression is governed by the following ordinary differential equations:

2.1 $$\frac{dU_{1}}{dt}=V_{1}\frac{1}{1+{\left(\phi_{L}L(t)+\sum_{j=1}^{N}a_{1j}U_{j}(t)/K_{ij}\right)}^{n}}-\gamma_{1}U_{1}(t)$$

and

2.2 $$\begin{array}{r} \frac{dU_{i}}{dt}=V_{i}\frac{1}{1+{\left(\sum_{\;j=1}^{N}a_{ij}U_{j}(t)/K_{ij}\right)}^{n}}-\gamma_{i}U_{i}(t)\;\text{for}\;i\in \{2,\ldots ,N\}, \end{array}$$

where the first and second terms on the right-hand sides correspond to the production and decay of the gene *i*, respectively. Parameter *V*_*i*_ is the basic production rate (i.e. the production rate without any inhibition) of gene *i*, 1/*K*_*ij*_ is the magnitude of inhibition of gene *i* expression by *j*, $\phi {\;}_{L}$ is the magnitude of inhibition of gene 1 by light, and *γ*_*i*_ is decay rate of gene *i*. The number of parameters was reduced by the non-dimensionalized setting *u*_*i*_(*t*) = *γ*_*i*_*U*_*i*_(*t*)/*V*_*i*_ and *ϕ*_*ij*_ = *V*_*j*_/(*γ*_*i*_*K*_*ij*_) [17]. Here, we focused on the evolution of the network structure and did not consider the variation in parameters. Thus, we set $\phi_{ij}=\phi_{L}=\phi$ for all *i* and *j*, meaning that the strengths of all regulations are equal. Note that gene *j* has the same impact as light on the expression of gene 1, when *a*_*ij*_ = 1 and *u*_*j*_(*t*) = 1. The final form of the system is described as

2.3 $$\frac{du_{1}}{dt}=\frac{1}{1+{\left[\phi \left(L(t)+\sum_{j=1}^{N}a_{1j}u_{j}(t)\right)\right]}^{n}}-u_{1}(t)$$

and

2.4 $$\frac{du_{i}}{dt}=\frac{1}{1+{\left(\phi \sum_{j=1}^{N}a_{ij}u_{j}(t)\right)}^{n}}-u_{i}(t)\;\text{for}\;i\in \{2,\ldots ,N\}.$$

To grasp the basic properties of this system, suppose that there is a simple GRN that has no inhibitory relationships (*M* = 0 and thus *a*_*ij*_ = 0 for all *i* and *j*), although it is an example of a subdivisible network. Under constant darkness, *L*(*t*) in equation (2.3) can be replaced with zero and thus $du_{i}/dt=1-u_{i}(t)$ for all *i*. We find a uniquely globally stable stationary state $u_{D}^{*}=(1,1,\ldots ,1)$, in which every gene shows a constant expression level of 1. Similarly, under constant light exposure, there is a globally stable stationary state $u_{L}^{*}=(1/(1+\phi^{n}),1,\ldots ,1)$ in which the expression of gene 1 is suppressed to 1/(1 + *ϕ*^*n*^). Under any given periodic LD cycle, the expression level of gene 1 fluctuated within the range of 1 and 1/(1 + *ϕ*^*n*^) with the same period as that of the LD cycle.

In the following numerical simulations, we took an hour as unit time and substituted *N* = 10, *M* = 20, *ϕ* ($=\phi_{L}=\phi_{ij}$) =100, *n* = 3, and a period of LD cycle (*τ* in appendix B) as 24, unless otherwise mentioned. A numerical solution up to *t* = 1248 (52 days) was obtained using Mathematica 11.2 (Wolfram Research Inc.). The initial values were set as *u*_*i*_(0) = 0 for all *i* ∈ {1, …, *N*}.

### (b) Classification of gene regulatory networks

We developed the following procedure to classify a GRN into three types: self-sustained oscillator, damped oscillator and hourglass. The first step was to discriminate between the self-sustained oscillator and the other two types based on convergence to a fixed point. The former and latter should not and should converge to a fixed point after a long time, and thus a standard test for convergence was applied. We measured the distance in the phase space $u=\{u_{1},u_{2},\;\ldots ,u_{N}\}$, between the states of the system at two time points *t*_*a*_ and *t*_*b*_ by Δ(*t*_*a*_, *t*_*b*_) = max _*i*_|*u*_*i*_(*t*_*a*_) − *u*_*i*_(*t*_*b*_)|. We classified systems that satisfy

2.5 $$\Delta (1200-\epsilon ,1200)>\delta_{1},$$

where *ε* = 10^−2^ and *δ*_1_ = 10^−6^, as candidates for a self-sustained oscillator. To check the regular periodicity of the candidate and exclude chaotic behaviour, we carefully examined the oscillation period *T*, which satisfies *u*(*t*) = *u*(*t* + *T*). The gene expression profiles that showed most frequent peaks from the 41st day to the 50th day was chosen among the *N* genes. Suppose that we found *n*_*p*_ peaks for the profile and denote times of those peaks as $t_{1}<t_{2}<\ldots <t_{n_{p}}$. We assumed that the system at time *t*_*k*_ remained in the vicinity of $u(t_{n_{p}})$ if *t*_*k*_ satisfied

2.6 $$\begin{array}{rl} & \max\{\Delta (t_{n_{p}}-12,t_{k}-12),\Delta (t_{n_{p}}-6,t_{k}-6),\Delta (t_{n_{p}},t_{k}),\\ & \;\Delta (t_{n_{p}}+6,t_{k}+6),\Delta (t_{n_{p}}+12,t_{k}+12)\}<\delta_{2}, \end{array}$$

where *δ*_2_ = 10^−4^. Denoting the greatest peak time among $t_{1},\;\ldots ,t_{n_{p}-1}$ that satisfies equation (2.6) as $t_{v}$, we obtained the oscillation period as $T=t_{n_{p}}-t_{v}$. If none of the peaks satisfies equation (2.6), we regarded the period of oscillation as ‘more than 10 days or chaotic.’

The second step for a system classified as a non-self-sustained oscillator is to discriminate between whether it was a damped oscillator or an hourglass. We regard $\hat{u}=(u_{1}(1200),u_{2}(1200),\ldots ,$ $u_{N}(1200))$ as a stable fixed point, and numerically obtained the leading eigenvalue of Jacobian matrix around the fixed point. The system is classified as a damped oscillator if the leading eigenvalue is a complex number and hourglass otherwise (see [9]). A similar procedure was applied to a GRN under LD cycles. The GRN under this condition is classified as a driven oscillator if equation (2.5) was satisfied. For driven oscillators, equation (2.6) was applied to estimate the period length of the driven oscillation.

### (c) Optimization using evolutionary algorithm

We applied an evolutionary algorithm to obtain a 10-gene-20-regulation network in which gene 10 shows an expression profile close to a given ideal profile. A cost value was numerically calculated using the following temporal integration:

2.7 $$\int_{49}^{50}{\left(f(t)-g(t)\right)}^{2}dt,$$

where *f*(*t*) and *g*(*t*) are the ideal and actual profiles of gene 10, respectively. One generation consists of mutation and selection. In the mutation process, we generated an offspring GRN by copying the parental GRN, deleting a randomly-chosen one of the *M* inhibitory relationships, and then added a new inhibitory regulation chosen from the *N*^2^ − *M* candidates (figure 1*c*). In the selection process, either the parental GRN or offspring GRN is selected as the parent in the next generation, following the rule below. The offspring is selected as the next parent if (i) it is a connected GRN (see appendix A), and (ii) it shows the same or smaller value of the cost function than that of the parent. Otherwise, the current parental GRN is selected as the next parent. For each set of an ideal profile and an environment, we ran 1000 independent trials with different initial parental GRNs and lasting 1000 generations.

### (d) Classification of networks based on bifurcation

We varied the value of the parameter *ϕ* in the evolutionarily optimized self-sustained oscillators to detect a supercritical Hopf bifurcation. We regarded that a system showed a supercritical Hopf bifurcation when the oscillation amplitude at steady-state continuously decreased to zero and period length did not change significantly as we reduced the value of *ϕ*.

## 3. Results

### (a) Self-sustained oscillation is rarer than damped oscillation and hourglass

**Figure 2.**
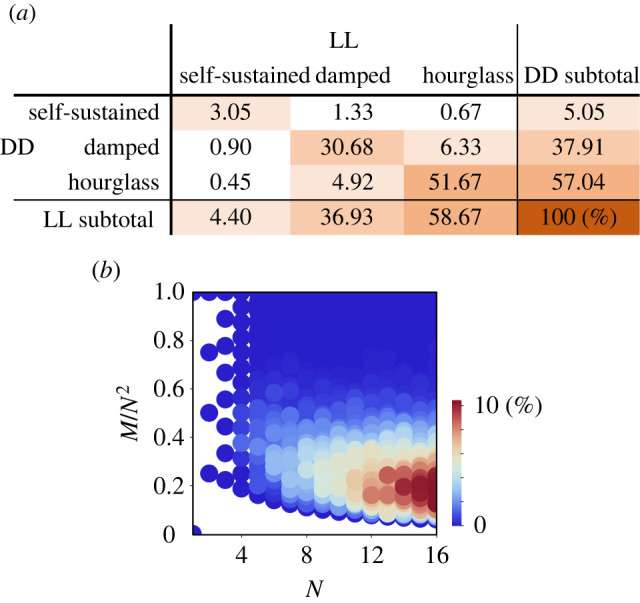
Dynamics of the randomly generated gene regulatory networks (GRNs). (*a*) Frequencies of the classes in one million connected GRNs consisting of 10 genes and 20 inhibitory relationships (i.e. *N* = 10 and *M* = 20). (*b*) Heat map indicating frequencies of self-sustained GRNs plotted as the number of genes (*N*) versus the filling rate of inhibitory relationships (*M*/*N*^2^). Note that a *N*-gene network can have at most *N*^2^ inhibitory relationships, including self-to-self inhibitions. For each pair of *N* (∈{2,…,16}) and *M* (∈{1, …, *N*^2^})), we test all possible GRNs if the number of possible GRNs (*N*^2^)!/[(*N*^2^ − *M*)! · *M*!] was less than 1000, or 1000 randomly generated GRNs otherwise. (Online version in colour.)

We first confirmed that the self-sustained oscillators were rarer than the other two types. Specifically, we randomly generated GRNs consisting of 10 genes and 20 intergenic relationships (i.e. *N* = 10 and *M* = 20) to obtain 1 000 000 connected GRNs. To complete the process above, we generated 1 131 417 GRNs because subdivisible GRNs were generated 131 417 times. We also found that very few (123) GRNs were generated twice, and no GRNs were generated more than twice. Only 6.4% of the GRNs exhibited self-sustained oscillations under either constant light (LL) or constant dark (DD) conditions (figure 2*a*). GRNs showing damped oscillation under at least one condition were found much more frequently (44.2%). The majority of GRNs (51.7%) was hourglasses under both constant conditions. GRNs that showed self-sustained oscillation under DD were slightly more frequent than those under LL. This asymmetry between LL and DD could be owing to our assumption that the expression level of gene 1 is kept near zero under LL. Effectively, this reduces the number of network member genes (*N*) from 10 to 9 (see below and figure 2*b* for the effect of *N*). In addition, the diagonal elements in figure 2*a* have greater values than expected under the assumption that behaviours under LL and DD were completely independent.

We then simulated a 12 h light/12 h dark environment (hereafter denoted as 12L12D) and confirmed that all GRNs exhibited oscillations with the period of 24 h. With such a setting, the gene expression dynamics under dark and light conditions are governed by different systems with different values (0 in the dark and 1 in the light; the value 1 means that the inhibitory effect from light on gene 1 is as strong as the possible maximum effect from one gene to other gene) for parameter *L*(*t*), and thus showed different sets of fixed points and even different oscillatory modes (figure 1*b*; electronic supplementary material, S1). Even a GRN behaving as an hourglass under both DD and LL conditions behaved as a daily oscillator because gene expression dynamics were periodically switched between two stable fixed points under dark and light conditions. We did not find any chaotic dynamics reported in previous studies [18–20]. This would stem from the difference that the light signal activates and suppresses expression of a gene in the previous and the present studies, respectively. Note that the numbers of fixed points under the LL and DD are not generally different, i.e. a fixed point under LL often has a corresponding one under DD. However, the stabilities of the fixed points differ owing to Hopf bifurcation or transcritical bifurcation when the bifurcation point for parameter *L* exists between zero and one [9].

**Figure 3.**
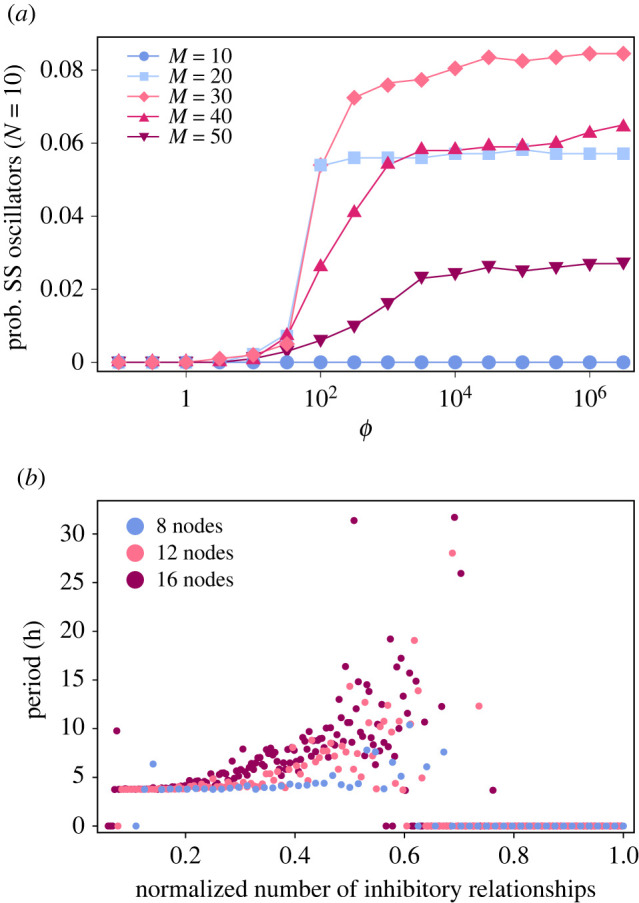
Dependency of properties of a randomly generated gene regulatory network (GRN) on parameters. (*a*) Proportion of GRNs showing self-sustained (SS) oscillation under dark versus *ϕ*. (*b*) Median period of self-sustained GRNs under dark versus normalized number of inhibitory relationships. Period zero means that no self-sustained GRNs were generated within 1000 trials. (Online version in colour.)

In addition, we examined the dependency on the number of genes *N* and on the number of edges *M*. The greater *N* and *M*, that is, a larger scale or a higher complexity of a GRN, favours self-sustained oscillation (figure 2*b*). Strong gene-to-gene suppression (i.e. greater *ϕ*) also facilitates self-sustained oscillations (figure 3*a*). A self-sustained oscillator involving more genes and/or a moderate number of edges had a longer period on average (figure 3*b*).

### (b) Seasonal environment can favour self-sustainability

**Figure 4.**
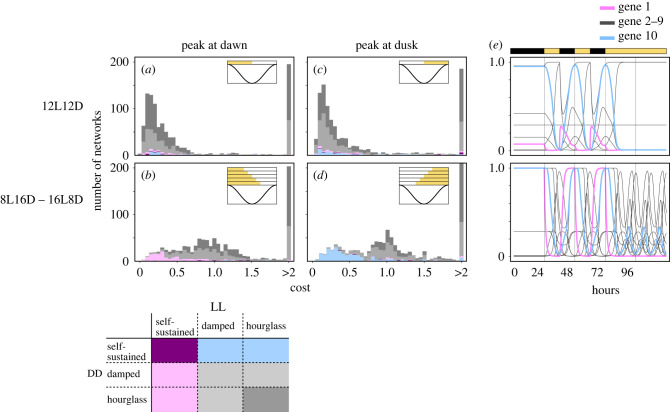
Results of evolutionary calculations in which a sinusoidal curve with a peak at dawn or dusk was assumed to be the ideal profile for gene 10 expressions. (*a*–*d*) Distribution of cost values for the outcome gene regulatory networks (GRNs) from 1000 different trials with different initial GRNs. The horizontal axes represent cost values, and the vertical axes indicate frequencies of GRNs. A cost value represents the deviation from the ideal profile. Histograms arranged in the left and right columns show outcomes of simulations in which the ideal expression profiles for gene 10 were sinusoidal curves with peaks at dawn and dusk, respectively. In conditions labelled as 12L12D (*a*,*c*), a GRN was repeatedly exposed to a 12 h light and 12 h dark cycle 50 times, and a cost value was calculated on the last 24 h. In conditions labelled as 8L16D–16L8D (*b*,*d*), a GRN was independently exposed to 8L16D, 10L14D, 12L12D, 14L10D and 16L8D environments, and cost values of those five environments were averaged. GRNs were classified according to behaviours under constant darkness and constant light exposure: purple for a GRN showing self-sustained oscillations under both constant environments, magenta and cyan for a GRN showing self-sustained oscillations only under light and dark conditions, respectively, dark grey if it does not show self-sustained or damped oscillations in any constant environment, and light grey otherwise. (*e*) Expression profiles of the genes in the GRNs with the lowest cost value in the 12L12D environment (top panel) and in the 8L16D–16L8D environment (bottom panel) when the ideal profile has a peak at dawn. Both GRNs were exposed to constant darkness for 24 h, the 12L12D condition for 48 h, and then under constant light. The GRN selected under the 8L16D–16L8D environment showed self-sustained oscillation under constant light.

**Figure 5.**
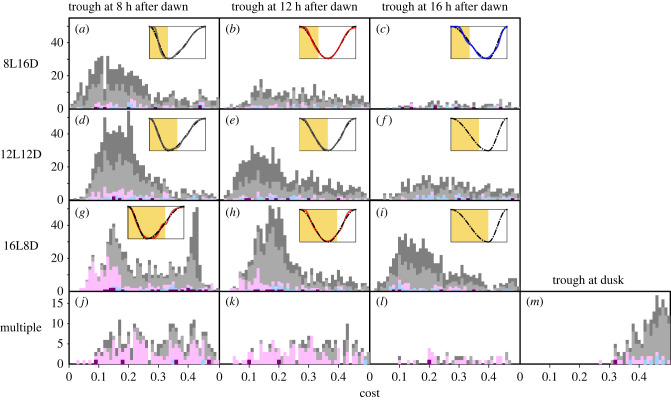
Distribution of cost values for the outcome gene regulatory networks (GRNs) from 1000 different trials with different initial GRNs under the cycles with different photoperiods. The horizontal axes represent cost values, and the vertical axes indicate frequencies of GRNs. A cost value represents the deviation from the ideal profile. Histograms arranged in the first, second and third columns show outcomes of simulations in which the ideal profiles were assumed to be sinusoidal-like curves with troughs at 8, 12 and 16 h after dawn, as shown by dotted-line curves in the embedded plots. Note that (*a*), (*e*) and ( *i*) can also be labelled as ‘trough at dusk’, which is the label for the fourth column. In the first, second and third rows, a GRN was exposed to 8 h light and 16 h dark (8L16D), 12L12D and 16L8D environments, respectively, repeatedly 50 times. Then a cost value was calculated on the last 24 h period. In the bottom row, a GRN was independently exposed to 8L16D, 10L14D, 12L12D, 14L10D and 16L8D environments, and cost values of those five environments were averaged. GRNs were classified according to behaviours under constant darkness and constant light exposure: purple for a GRN showing self-sustained oscillations under both constant environments, magenta and cyan for a GRN showing self-sustained oscillations only under light and dark conditions, respectively, dark grey if it does not show self-sustained or damped oscillations in any constant environment, and light grey otherwise. Coloured curves in the embedded plots are expression profiles of gene 10 in the GRNs with the lowest cost values. Note that panels (*e*) and (*k*) are rescaled duplications of figure 4*a* and 4*b*, respectively.

To specify selective force(s) that can lead to a non-random evolution of self-sustained oscillation, we performed our evolutionary algorithm and collected the 10-genes networks under some LD conditions in which the expression of gene 10 had an ideal profile. We specified environmental conditions that facilitated the self-sustainability by observing the type of the GRNs obtained through the evolutionary algorithm. The best GRN in an environment was defined as the GRN with the smallest cost value among outcomes of 1000 trials of the evolutionary computation. We examined multiple ideal profiles one by one. Some of those ideal profile depended on day length similarly to gene 1, and the others were independent from day length (electronic supplementary material, figure S3*a*). Throughout the series of simulations described in this section and in figures 4, 5; electronic supplementary material, S3, we premised a strong inhibitory effect from light to gene 1 for all GRNs as in the previous section, which made gene 1 profile photoperiod-dependent. With this premise, we considered the networks that had at least one photoperiod-dependent gene.

First, assuming a 24 h periodic sinusoidal curve with a peak at dawn as the ideal profile, we performed evolutionary computation under the 12L12D environment. A majority of the final outcomes showed self-sustained oscillation neither under dark nor under light (figure 4*a*; the top panel of figure 4*e*). Using a sinusoid curve with a peak at dusk as the ideal profile resulted in a similar distribution. (figure 4*c*).

We next considered a seasonal environment mimicking a temperate zone. Specifically, the cost value of a GRN was defined as the average of five cost values independently calculated under the 8L16D, 10L14D, 12L12D, 14L10D and 16L8D environments. In the evolutionary computation, we applied the same sinusoidal curve with a peak at dawn, yielding a trough 12 h after dawn, to the ideal profile for gene 10 regardless of the day-length environment. This setting corresponds to the external coincidence model for photoperiodism (see Discussion). With this setting, most GRNs with lower cost, that is, higher fitness, behaved as self-sustained oscillators under the constant light condition while damped oscillators or hourglasses under the constant dark condition (figure 4*b*, bottom panel of figure 4*e*). In addition, the final outcomes had on average greater cost values than those in the previous computation with the single 12L12D environment (compare figure 4*a*,*c* with 4*b*,*d*), indicating the difficulty of achieving similar expression profiles under multiple light environments. Changing the peak time of the curve from dawn to dusk resulted in qualitative alterations in the outcomes; self-sustained oscillators only under dark was frequently observed in place of self-sustained oscillators only under light (compare figure 4*b* and 4*d*).

Of the GRNs optimized through evolutionary computation under seasonal environment, those showing self-sustained oscillations under light (magenta or purple cases in figure 4*b*) were further analysed to determine how they lost their self-sustainability (i.e. which type of bifurcation they followed) when the parameter of the system *ϕ* was reduced from 100. Most (161 of 164) GRNs transitioned from self-sustained oscillators to non-oscillators via supercritical Hopf bifurcation (electronic supplementary material, figure S2*a*), and the other three GRNs appear to show other types of bifurcation (electronic supplementary material, figure S2*b*).

We tested the generality of the above-obtained conclusion that self-sustained and non-self-sustained oscillators have higher fitness than the other types under seasonal and aseasonal environments, respectively. Changing the trough time of the ideal profile from 12 h after dawn (the sinusoidal curve in figure 5*b*,*e*,*h*) to 8 or 16 h after dawn (the distorted sinusoidal-like curves in figure 5*a*,*c*,*d*,*f*,*g*,*i*) did not qualitatively change this conclusion (figure 5). It was clarified that self-sustained oscillations are especially useful to revert the expression level of gene 10 at midday (figure 5*g*). Though 16L8D environment can facilitate evolution of self-sustained oscillators under some conditions (figure 5*g*,*h*), it is not a general tendency (figure 5*i*) unlike seasonality (figure 5*j*–*l*). In addition, we considered the case that ideal profile is dependent on day length. Specifically, we applied the asymmetric sinusoidal-like curve with a peak at dawn and a trough at dusk (electronic supplementary material, figure S3*a*). Evolutionary computation using this flexible wave form yielded lower frequencies of self-sustained oscillators among the outcomes compared with that using the sinusoidal profile (electronic supplementary material, figure S3*b*; compare figure 5*a* with 5*b* and figure 5*i* with 5*h*). The same pattern was observed in the evolutionary computation assuming a seasonal environment; less self-sustained oscillators and more damped oscillators or hourglasses were included in the high fitness group when the day-length dependent form was applied than when fixed wave form was applied (compare figure 5*m* with 5*j–l*). We also perturbed the function form of the ideal profile by sharpening the peaks and flattening the troughs (the bluish and reddish curves in the electronic supplementary material, figure S3*c*, respectively). Such a form is modelled on circadian gating, which realizes a long inactive or insensitive phase often through regulation by the circadian clock [21]. It turned out that self-sustained oscillators can appear even in aseasonal conditions when the gating-type profile is optimal (see the electronic supplementary material, figure S3*d*). Regarding seasonal conditions, our evolutionary computation did not detect any GRNs with high fitness (electronic supplementary material, figure S3*e*).

## 4. Discussion

This study examined the evolution of self-sustainability in the circadian clock by observing the behaviour of random GRNs under diurnal cycles. We found that GRNs showing self-sustained oscillation under a constant light condition are rarer than those losing self-sustainability, that is, those showing damped oscillation or hourglasses. However, GRNs with self-sustainability showed higher fitness than the other types in evolutionary computation when they had to generate an expression profile with a constant form regardless of day length (figure 4*b*,*d*), whereas a non-self-sustained system was preferable when the systems could use the environmental switch, that is, when the ideal gene expression showed a trough at dusk (figure 4*a*,*c*). These results suggest that seasonal variation of day length is a driving force of evolution of self-sustainability.

To shape a specific non-flat profile, a non-self-sustained oscillator has to largely depend on an external force. It follows that different forms of the external force, i.e. different day lengths, impose largely different profiles. On the other hand, a self-sustained oscillator can take another strategy of making its intrinsic profile close to an ideal one and reducing influence by external force. Self-sustained oscillators are thus advantageous in forming a constant non-flat profile regardless of day length. The above mechanism is essentially the same as features focused on in previous studies that non-self-sustained and self-sustained oscillators are fragile and robust to external noise, respectively [22,23]. More precisely, self-sustainability under constant light contributes to the realization of a trough under light period in a cycle (figure 5*a*,*g*,*b*,*h*). Moreover, the trough at midday was more effective (compare figure 5*g* with 5*h*). These results suggest that self-sustainability in LL conditions can contribute to both peaks and troughs during the light period of LD cycles. In other situations, a damped oscillator or hourglass may work as an alternative to a self-sustained clock. The fact that shifting the peak time to the beginning of the dark period accelerates the preference for self-sustainability in DD supports this hypothesis (figure 4*d*). Thus, a higher latitude can benefit self-sustainability because seasonal conditions necessarily contain this self-sustained-dominant condition.

We also found that GRNs with the property of self-sustainability were exclusively favoured to generate an identical gene expression profile regardless of the day length (figure 5*j–l*; note that every one of those GRNs consisted of both photoperiod-dependent (e.g. gene 1) and photoperiod-independent (e.g. gene 10) profiles), whereas damped oscillators and hourglasses seemed to have difficulty generating a consistent profile independent of day length. The endogenous season-independent timing system is implicitly presumed by the external coincidence model, stating that a seasonal event (e.g. flowering or hibernation) is launched when the photoperiod coincides with the endogenous rhythm [24]. In the example of *Arabidopsis* flowering, the CONSTANS (CO) protein is known to induce floral transition when its amount is above a threshold and to be degraded under dark. In addition, CO gene expression is regulated by the circadian clock. Given that the expression profile of CO is not greatly affected by day length and reaches above the threshold at a certain timing, say 16 h, flowering is and is not induced when the day length is longer and shorter than 16 h, respectively [25]. There have been so far, few empirical evidences of the external coincidence model, and future theoretical studies should specify a type or function of genes that are more likely to show a photoperiod-independent profile.

Our finding that seasonality can favour the evolution of self-sustainability is consistent with comparative studies on the variation in circadian rhythms across latitudes. *Synechococcus*, a cyanobacterial genus known to have a circadian self-sustained oscillator, is widely distributed both under seasonal and aseasonal environments. By contrast, *Prochlorococcus*, a genus with a light-driven 24 h oscillator, are absent in high-latitude regions with strong seasonality [26]. Previous theoretical studies focusing on the circadian machinery in these two genera deduced another explanation concerning noise sensitivity [22,23], which is not mutually exclusive with our seasonality hypothesis. Using duckweed species in the genus *Lemna* distributed between tropical and subarctic zones, Isoda *et al.* [27] found a tendency similar to the above. They examined the self-sustainability of the circadian rhythm of plants in a wide range of temperature conditions and reported that the species inhabiting colder (i.e. higher latitudinal) regions had more stable self-sustainability than those inhabiting lower latitudinal regions, indicating the importance of self-sustainability in seasonal environments and/or non-necessity of self-sustainability in aseasonal environments. We believe that this paper bridges chronobiology and ecology more strongly and thus expect other undescribed examples of organisms lost their self-suitability in tropical areas.

Self-sustained oscillation is more likely to appear in larger-scale and more-strongly-connected GRNs involving many members and a moderate number of regulatory relationships (figures 2*b* and 3*a*). This may correspond to the fact that self-sustained circadian clocks are generally large-scale. The conceptual model of the *Drosophila* circadian clock provided by Rivas *et al*. [28] involved 12 genes. Sanchez & Kay [29] states that ‘minimal architecture’ of the circadian clock in *Arabidopsis thaliana* consists of 10 genes. Increased number of genes may also contribute to a sufficiently long period of self-sustained oscillation (figure 3*b*). Another way to develop a longer-period oscillation is to have more gene-to-gene regulation relationships (figure 3*b*), though self-sustained oscillation itself may not occur in GRN with too many links (figure 2*b*).

The perturbation of the parameters of the system in the optimized GRNs mostly caused supercritical Hopf bifurcation (electronic supplementary material, figure S2), meaning that the oscillation amplitude, rather than the period, depends more on the parameter. The preference for Hopf bifurcation in our algorithm suggests that circadian clocks in nature can be surrounded by a damped oscillator region in the parameter space. Lowering the ambient temperature nullifies cyanobacterial circadian clocks via Hopf bifurcation [30]. This result contrasts with the fact that the period of most GRNs obtained via Kobayashi’s algorithm is sensitive to *ϕ* [17]. This robustness against perturbation at parameter suggests that circadian rhythms are not merely self-sustained but also share other common properties embedded in network topology. Further research would hint at the enigma in chronobiology: why transcriptional and translational feedback loops has been detected in all of the circadian clocks including cyanobacteria that possess post-translational oscillator [31–34].

One limitation of the present study is that our research considered GRNs with only nonlinear inhibitory regulations [17]. This model is relatively abstract; however, studies using other models (e.g. the Boolean network model [35]) are required to exclude the model dependency of our results. It is also required to incorporate direct activation links, which are known to exist in real circadian systems [36] and are theoretically suggested to help generate self-sustained oscillation in some situations [37]. Another limitation is the use of a simple evolutionary algorithm, in which one gene-to-gene regulatory relationship dissolves and a novel relationship appears. A real biological mutation would alter magnitude of the existing regulation (the value of *ϕ*_*i*_ in the model) or enlarges/shrinks the scale (i.e. the number of genes involved) of the GRN. Thus, a more complex mutation system is required to trace the evolutionary pathway of the circadian clock more precisely. In addition, the use of an evolutionary algorithm could result in relatively few GRNs showing a gene expression profile that is highly similar to an ideal profile. A more efficient method, such as the Markov chain Monte Carlo method [38], would provide a greater number of good-fit GRNs, which would help to understand selective force on circadian clocks. Increased number of GRNs showing self-sustained oscillation would in turn shed light on another interesting topic: which topological characteristics of GRNs (e.g.frequency and positions of autoregulatory pathways) are related to the property of self-sustained oscillation. This will become possible with a novel method to systematically process and analyse hundreds of thousands of networks. Further studies are clearly needed.

There have been few reports on the evolution of self-sustained circadian rhythms. Laboratory evolution assays are straightforward for this topic; however, no reports have succeeded in demonstrating the appearance of the circadian clock in the laboratory. Drawing a phylogenetic tree based on genome sequences can provide a history of circadian molecular machinery, but not the adaptive mechanism. Instead, we have numerically introduced a tractable approach based on an evolutionary algorithm, which provided an unbiased finding that seasonality facilitated the evolution of the self-sustained circadian clock. We expect that confirmation of our idea by experiments and further theoretical studies should advance the study of the evolution of circadian rhythms.

## Data Availability

Code implementing the model of gene regulatory network (GRN) and searching for the optimal GRN is available on Github https://github.com/hito1979/RSB2022. The data are provided in the electronic supplementary material [39].

## Appendix A. An algebraic method to check subdivisibility of networks

A network is considered connected if it contains at least a one-way path between every pair of nodes. It is called strongly connected if it includes at least one path directed from every node to another. A network with *N* nodes represented by an adjacency matrix M is strongly connected if ${\left(I+M\right)}^{N-1}$ is a positive matrix, where I is an *N* × *N* identity matrix [40].

To check whether a graph represented by an adjacency matrix A is connected, we define a new *N* × *N* matrix $B=(b_{ij})$, where

$$b_{ij}=\max\{a_{ij},a_{\;ji}\}.\;(A\;1)$$

The focal network is connected if the network represented by B is strongly connected, that is, if the matrix ${\left(I+B\right)}^{N-1}$ is positive.

## Appendix B. Continuation of the periodic binary function

Because the method used in this study to obtain numerical solutions for differential equations can cause a numerical error in analysing a system involving a sudden or unsmooth change in a variable, it is convenient to use smooth and continuous functions. The master equations describing the proposed model (equations (2.3) and (2.4)) contain a discontinuous function, *L*(*t*), to represent periodic change in the light status. By revising a function previously proposed by Pokhilko *et al.* [41], we approximated *L*(*t*) using the following function:

$$L(t)=\frac{\;f\;(t_{R})-f\;(t_{S})+f\;(\tau +t_{R})}{2}\;(B\;1)$$

and

$$f\;(x)=1+\tanh\frac{t-\tau \cdot \mathrm{floor}(t/\tau )-x}{\epsilon },\;(B\;2)$$

where $t_{R}$ and $t_{S}$ are the phases of sunrise and sunset, respectively; *τ* is the period of an LD cycle; and *ε* is the duration of the transition between 0 and 1. We have used *τ* = 24 and *ε* = 10^−2^.
